# Hedgehog Signaling Pathway in Ovarian Cancer

**DOI:** 10.3390/ijms14011179

**Published:** 2013-01-09

**Authors:** Joanna Szkandera, Tobias Kiesslich, Johannes Haybaeck, Armin Gerger, Martin Pichler

**Affiliations:** 1 Division of Clinical Oncology, Department of Medicine, Medical University of Graz, 8036 Graz, Austria; E-Mails: joanna.szkandera@medunigraz.at (J.S.); armin.gerger@medunigraz.at (A.G.); 2 Department of Internal Medicine I, Paracelsus Medical University/Salzburger Landeskliniken (SALK), 5020 Salzburg, Austria; E-Mail: t.kiesslich@salk.at; 3 Institute of Pathology, Medical University of Graz, 8036 Graz, Austria; E-Mail: johannes.haybaeck@medunigraz.at

**Keywords:** Hedgehog pathway, ovarian cancer, carcinogenesis, targeted therapy

## Abstract

Despite advances in surgical and chemotherapeutic treatment options, less than 50% of patients with advanced-stage ovarian cancer survive five years after initial diagnosis. In this regard, novel treatment approaches are warranted utilizing molecularly targeted therapies directed against particular components of specific signaling pathways which are required for tumor development and progression. One molecular pathway of interest is the hedgehog (Hh) signaling pathway. Activation of the Hh pathway has been observed in several cancer types, including ovarian cancer. This review highlights the crucial role of Hh signaling in the development and progression of ovarian cancer and might lead to a better understanding of the Hh signaling in ovarian tumorigenesis, thus encouraging the investigation of novel targeted therapies.

## 1. Introduction

Ovarian cancer is the fifth most lethal gynecologic malignancy in the Western world with an estimated 22,280 new cases and 15,500 deaths in 2012 in the United States alone [1]. Epithelial ovarian cancer accounts for over 90% of all ovarian malignancies and comprises five histological subtypes: serous, mucinous, endometrioid, undifferentiated and clear cell type. The remainder arises from germ cells or stromal cells [2]. Due to the lack of appropriate tumor markers and of clinically significant symptoms at early tumor stages, most patients are diagnosed at advanced stage III or IV [2]. The clinical behavior of this malignancy varies from an excellent prognosis and high probability of cure, to rapid progression and poor prognosis, reflecting variation in the tumors’ biological properties [2]. Despite good responses to the initial treatment, which involves surgery followed by chemotherapy, the five-year relative survival rate is only 44% [1]. The major unresolved clinical problems associated with ovarian cancer include malignant progression and rapid emergence of drug resistance against conventionally used chemotherapeutic agents.

Currently, the etiology of ovarian cancer is still not fully understood. It is generally accepted that ovarian carcinoma arises from ovarian surface epithelium (OSE), a primitive mesothelium with both epithelial and mesenchymal characteristics [3]. The ovarian surface epithelium has the ability to differentiate diversely in response to different stimuli [3]. Ovarian cancer displays a unique characteristic: in contrast to other cancers, during carcinogenesis, it usually becomes more differentiated than the epithelium from which it originates and shows an increased E-cadherin expression upon progression [4].

The hedgehog (Hh) signaling pathway was initially discovered in 1980 through genetic analyses of segmentation of the *Drosophila* fruit fly [5]. In humans, the Hh pathway was identified to function as a key mediator of many fundamental processes during embryonic development. In more detail, the Hh pathway is involved in regulating differentiation and proliferation, including cell fate and number, in brains and spinal cords, and the pattern of limbs and internal organs, so that developing tissue reaches its correct size with the appropriate cell types and adequate degrees of vascularization and innervation. The Hh pathway even controls body heights as well as regulates aging and its associated chronic degenerative and inflammatory diseases [6–9]. Furthermore, Hh signaling promotes proliferation, regeneration and differentiation of somatic tissues in adults [6]. It also plays a pivotal role for maintaining the tissue stem cell population [10]. Inactivation of this pathway contributes to hereditary developmental defects such as holoprosencephaly, whereas hyperactivation of this pathway by mutations is found in human cancers, such as medulloblastoma, basal cell carcinoma (BCC) and extracutaneous cancers [11–14]. In this article, we give an overview regarding the signal transduction of the Hh pathway and discuss the role of Hh signaling in development and progression of cancer with special emphasis on ovarian cancer and its potential impact on future therapeutic strategies.

## 2. Signal Transduction of the Hedgehog Pathway

**Figure 1:**
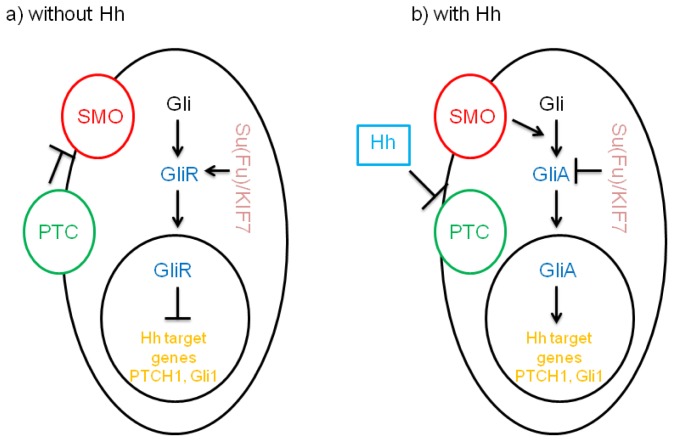
Overview of the Hedgehog signaling pathway: a simplified model for Hh signaling in mammalian cells. SMO is the key transducer of the Hh pathway. (**a**) In the absence of the Hh ligands, the putative Hh receptor PTC is localized in the cilium and inhibits SMO signaling. Gli molecules are processed with the help of Su(Fu)/KIF7 molecules into repressor forms, which deactivate the Hh signaling pathway. (**b**) In the presence of Hh, PTC is displaced out of the cilium and unable to inhibit SMO. Hh reception facilitates conformational changes in SMO, promoting Gli activation (GliA) and stimulation of Hh target gene expression. Su(Fu) and KIF7 can inhibit this process.

The general signaling mechanisms of the Hh pathway are conserved from insects to humans and are illustrated in Figure 1 [15]. Three Hh homologs with different spatial and temporal distribution patterns have been identified in humans in the early 1990s: Sonic hedgehog (Shh), Indian hedgehog (Ihh) and Desert hedgehog (Dhh) [16–18]. Following translation, the Hh protein precursor enters the secretory pathway and undergoes autoprocessing and lipid modifications to release its *N*-terminal fragment (HhN), which is bound to a cholesterol moiety at the *C*-terminal end [19–21]. Several molecules are involved in the reception of Hh ligands, with Patched (PTC, one PTC in fly and two PTC in vertebrates—PTCH1 and PTCH2) representing the major receptor [22]. Hh binding to the PTC on the target cell initiates the Hh signaling cascade [23]. The Hh-interacting protein (HIP) competes with PTC to bind Hh, leading to a negative regulation of Hh signaling [24]. In the absence of the Hh ligand, PTC represses the activity of Smoothened (SMO), the seven transmembrane domain-containing protein, which serves as the key signal transducer [25]. However, it is still not clear how the binding of Hh proteins results in the pathway activation. One hypothesis is that PTC inhibits the function of SMO in the absence of Hh by transporting small endogenous molecules specifically targeted to SMO, such as PI4P, lipoproteins, and pro-vitamin D3 [26–28]. Binding of Hh to PTC shuttles PTC out of the cilium, so that it is no longer able to inhibit SMO, resulting in downstream inhibition of molecules by SMO signaling. There are two important events during mammalian SMO signaling. First, the SMO protein passes through conformational changes to favor intermolecular interaction of SMO [29]. Second, translocation of mammalian SMO to cilia plays an important role during Hh signaling. The function of primary cilium is regulated by large protein complexes involved in intraflagellar transport (IFT), which functions in anterograde and retrograde movement of cargo within the primary cilia [30]. Several Hh components, including SMO and Gli molecules, are also present at the primary cilium, highlighting the impact of cilium in Hh signaling [31,32]. As a transcription factor, Gli protein can regulate target gene expression by association with specific consensus sequences located in the promoter region of the target gene [33]. It has been reported that SMO mutants lacking a ciliary translocation signal fail to mediate Hh signaling [34]. However, recent data indicate the translocation of SMO to cilium as not being sufficient to activate Hh signaling [32,35]. Using tissue-specific gene knockout, recent studies demonstrate dual roles of cilium in Hh signaling-mediated carcinogenesis in a mouse model by knocking out the cilium component Kifa3 [32,36]. Whereas Kifa3 is required for activated SMO-mediated tumor formation, deletion of Kifa3 catalyzes Gli2-mediated carcinogenesis. In *Drosophila*, several molecules, including COS2 and Fused (Fu), have been identified downstream to SMO signaling, but it has to be elucidated how their vertebrate homologs function in Hh signaling. Analysis of the function of vertebrate homologs of COS2, KIF7 and the closely related KIF27 in cultured mammalian cells has led to the conclusion that neither protein has a role in Hh signaling [37]. However, recent *in vivo* studies suggest a role for Kif7 in coordinating Hh signal transduction in mice, yet no direct interaction between SMO and KIF7 has been detected, thus indicating that the function of COS2 in vertebrates is replaced by other molecules [38]. In Fu null mice, no changes of Hh signaling have been observed, indicating that Fu is not critical for Hh signaling during embryonic development of vertebrates [39]. In mammalian cells, several novel cytoplasmic regulators of Hh signaling have been discovered, including Rab23 and tectonic [40,41]. Both are negative regulators of Hh signaling situated downstream of SMO. Rab23 is localized in the nucleus as well as in the cytoplasm, implying other yet unknown functions apart from membrane trafficking [42]. Recent data suggest that Suppressor of Fused (Su(Fu)) operates as a tumor suppressor gene in mammalian cells. Su(Fu) was identified in *Drosophila* by its ability to suppress active fused mutations, but it is not required for the activity of the pathway. Su(Fu) null mouse mutants are inefficient in repressing the pathway and have some phenotypes similar to PTCH1 inactivation [43]. PTCH1^+/−^ mice develop medulloblastoma, rhabdomyosarcoma and basal cell carcinoma following irradiation, whereas Su(Fu)^+/−^ mice predominantly develop basaloid epidermal proliferations [44–46]. Loss of Su(Fu) results in the activation of Hh signaling, indicating a central role of Su(Fu) in the pathway repression [43]. At the molecular level, Su(Fu) was found to associate directly with Gli function and is essential for Gli3 processing [47,48]. Finally, Hh signaling activates downstream Gli transcription factors, known to regulate target gene expression by binding to a consensus binding site in the promoter of the target gene region [33,49,50]. Several regulatory feedback loops are found in the Hh pathway, maintaining the level of Hh signaling in cells. PTC and HIP provide negative feedback mechanisms. In contrast, Gli1 and GAS1 form positive regulatory loops. Alterations of these loops result in abnormal signaling of the Hh pathway, such as loss of PTCH1 in BCC.

## 3. Activation of the Hedgehog Pathway in Human Cancer

Significant progress in gaining knowledge about Hh signaling in human cancers was achieved by the discovery that mutations of the human homolog of the *Drosophila* patched gene (PTCH1) are associated with a rare hereditary form of BCC (basal cell nevus syndrome, also called Gorlin syndrome) [51,52]. Gorlin syndrome is a rare autosomal genetic disorder with two distinct sets of phenotypes: a high risk of BCC and a predisposition to develop medulloblastoma and developmental defects such as bifid ribs and ectopic calcification. The role of PTCH1 has been further demonstrated in knockout mice: mice heterozygous for PTCH1 null mutation showed essential features observed in patients with Gorlin syndrome, such as malignant tumors (medulloblastomas, rhabdomyosarcomas, basal cell carcinomas) and developmental defects, such as spina bifida occulta [45,46,53]. BCC, one of the most common human cancers, regularly shows abnormalities of the Hh pathway arising from mutations in PTCH1 (50%), SMO (10%) and other genes, including Su(Fu) [54,55]. About one third of medulloblastomas show activated Hh signaling, and mutations influencing the signaling cascade could be commonly detected within the PTCH1 gene. Increasing evidence supports the notion that the activation of Hh signaling plays an important pathophysiolocial role in many types of human cancer. It is postulated that over 30% of human cancers are presented with activated Hh signaling, including brain tumors, melanomas, leukemias, lymphomas, gastrointestinal, kidney, bladder, pancreatic, liver, prostate, lung, ovarian and breast cancers [56]. In those cancers, gene mutation is not primarily responsible for activated Hh signaling, but rather caused by ligand-dependent mechanisms or non-canonical Hh signaling activation [57,58].

## 4. The Role of Hedgehog Signaling in Cancer Initiation and Progression

Recent data indicate that Hh signaling is involved in different stages of carcinogenesis. In Barrett’s esophagus, an early precursor of esophageal adenocarcinomas, the expression of Shh and Ihh is increased in the epithelium, which is associated with stromal expression of the Hh target genes PTCH1 and BMP4 [59]. These results suggest that Hh signaling plays a significant role in the initiation of esophageal adenocarcinomas. Furthermore, activation of this pathway was observed in pancreatic intraepithelial neoplasia (PanIN) lesions as well in metastases of pancreatic cancer, suggesting that Hh signaling is also important for pancreatic cancer [60]. However, transgenic mice with pancreatic-specific expression of Shh or Gli2 develop undifferentiated pancreatic tumors which substantially differ from pancreatic ductal adenocarcinomas (PDAC), indicating that exclusive activation of Hh signaling is not sufficient to trigger PDAC development [61]. In gastric and prostate cancers, the activation of Hh signaling is associated with cancer progression [62–64]. In line with these observations, the inhibition of Hh signaling in prostate and gastric cancer cells reduces tumor cell invasiveness [63,65,66]. Increasing evidence also suggests that Hh signaling plays an important role in the development and progression of glioma, breast cancer, ovarian cancer, leukemia, and B-cell lymphoma [67–72]. The modes of Hh signaling in cancer development may vary from one tumor type to another. Several studies have indicated that in the absence of mutation in Hh pathway components, tumor cells synthesize and respond to Hh ligand in an autocrine-juxtacrine manner [57,65,73]. However, recent reports support an alternate model in which tumor-derived Shh or Ihh ligands trigger Hh signaling in the stromal environment in a paracrine manner [61,74]. It could be demonstrated that Hh pathway inhibition in pancreatic and colon carcinoma xenograft models blocks paracrine signaling between the tumor and its adjacent stroma [75]. A study in B-cell lymphomas proposes a different paracrine Hh signaling in which Shh is secreted by stromal cells to activate the Hh pathway in cancer cells [72,76]. It is noteworthy that correlation of Hh target gene expression with tumor specimens is not sufficient to clarify the role of Hh signaling in a specific cancer type. Establishing animal models using tissue specific activation of Hh signaling is crucial for understanding the role of Hh signaling in tumor progression. In an orthotopic model of pancreatic cancer, Yang *et al*. observed both paracrine and autocrine Hh signaling [77]. Increasing evidence indicates that Hh signaling is required for maintenance and function of cancer stem cell population. For example, leukemia stem cell maintenance and expansion was reported to be dependent on hedgehog signaling [78,79]. In the absence of SMO or treatment of cyclopamine, a specific inhibitor of the SMO co-receptor, the hematopoietic stem cell population is reduced. Based on cancer stem cell theory, it is assumed that Hh signaling activation may exert chemotherapy or radiotherapy resistance in cancer if this pathway has a major role in cancer stem cell functions [80]. Indeed, several studies have demonstrated that the activation of Hh signaling is associated with chemotherapy or radiotherapy resistance [81,82]. It was recently shown that the Hh signaling inhibitor IPI-926 enhances the delivery of the chemotherapeutical drug gemcitabine in a mouse model of pancreatic cancer [82]. Upon reviewing the literature on Hh signaling in human cancer, different results have been reported in different tumor types, and sometimes the results are contradictory to each other. These discrepancies are due to several reasons. First, it seems possible that the function of Hh signaling in human cancers may be context-dependent, occurring in some tissues or cell lines but not in others. For example, recent reports indicate that Hh signaling functions in maintaining cancer stem cell proliferation, but not in the proliferation of all cancer cells [78,79]. Second, the heterogeneity in tumor tissue is an essential factor in the analysis of Hh target gene expression by real-time PCR. For example, identification of activation of the Hh pathway in prostate cancer specimens can be obtained more frequently from transurethral resection of the prostate (TURP) specimens than from prostatectomy specimens [63]. Whereas the prostatectomy specimens contain only 5%–10% of tumor cells in the tissue, the TURP specimens generally have more than 70% of tumor cells. Therefore, the data from these two types of specimens may differ due to the percentage of cancer cells in each tissue. Third, different standards have been utilized to define Hh signaling activation. Some groups use elevated expression of Gli1 transcripts as the read-out of Hh signaling activation, whereas others examine the expression level of Hh target genes, such as Gli1, PTCH1, secreted frizzled-related protein 1 (sFRP1 ) and Hh-interacting protein (HIP) [64,65,68,83–85]. Similarly, some investigators apply exclusively immunohistochemistry to determine Hh signaling activation, whereas most studies use multiple approaches, including *in situ* hybridization, real-time PCR, and immunohistochemistry [71,86]. As the research in this area progresses, our knowledge about Hh-signaling activation in human cancer will also increase, accompanied by the development of appropriate methodological approaches [87].

## 5. Small Molecule Modulators of Hedgehog Signaling

**Figure 2:**
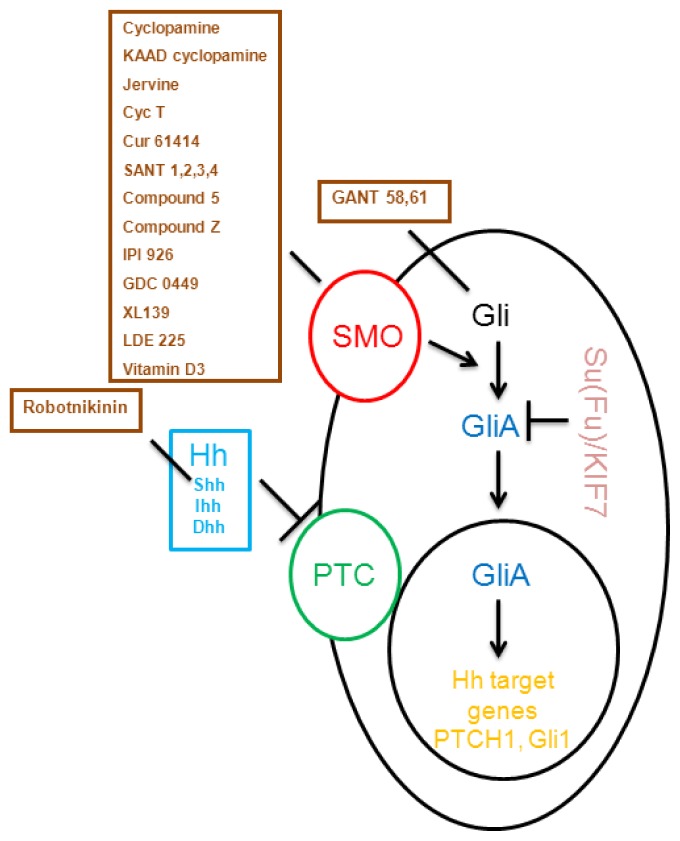
Overview about currently tested Hedgehog pathway inhibitors and their mode of action.

More than 50 compounds known to inhibit Hh signaling have been identified. Among these, five are used in clinical trials [88–97]. Three major targeting sites for Hh signaling have been determined: Hh molecules (Shh neutralizing antibodies, small molecule Robotnikinin), SMO protein (cyclopamine and its derivatives IPI-926, Cyc-T and synthetic compounds GDC-0449, Cur61414, XL-139, and LDE-225) and Gli inhibitors (HPI-1, HPI-2, GANT-56, and GANT-61). Hh signaling inhibitors can be divided into three groups: natural products (cyclopamine, its derivatives and other natural products), synthetic small molecules, and Hh signaling modulators. Table 1 lists the compound currently used in preclinical or phase I/II clinical trials, and Figure 2 gives an overview about the mode of action for some of these agents.

### 5.1. Natural Products and Analogs (Cyclopamine, Its Derivatives, and Others)

Cyclopamine is a plant-derived steroidal alkaloid which inhibits Hh signaling through direct binding to the transmembrane helices of SMO [97]. The discovery of specific, small molecule antagonists of SMO has established new options for the targeted therapy of human cancers associated with Hh signaling. The *in vivo* effect of cyclopamine on tumor shrinkage has been shown in several mouse models [98–100]. Cyclopamine derivatives with additional modifications aiming to increase acid stability and aqueous solubility are now available, such as IPI-926 and Cyc-T [94,104]. IPI-926 is now in use in clinical trials. In addition, several other synthetic compounds have been identified that directly inhibit SMO activity but with no structural similarity to cyclopamine [102,109].

### 5.2. Synthetic Hh Signaling Antagonists

Several synthetic Hh inhibitors have been reported in the literature, most of them directed to SMO (e.g., GDC-0449) and other compounds targeting other Hh signaling components, including Shh and Gli.

A clinical trial with vismodegib (GDC-0449) in a medulloblastoma patient resulted in a rapid reduction of tumor mass, but led to drug resistance due to SMO mutation [90,92]. Moreover, vismodegib was evaluated in a phase I, dose-escalation study of patients with refractory solid tumors. Activity was seen in patients with advanced BCC, medulloblastoma and other solid tumors [89–91]. A small-molecule inhibitor for Gli1 inhibited tumor cell proliferation *in vitro* and successfully blocked cell growth in an *in vivo* xenograft model using human prostate cancer cells harboring downstream activation of the Hh pathway [106]. Another small molecule called Robotnikinin was reported to bind Shh protein and to block Shh signaling in cell lines, human primary keratinocytes, and a synthetic model of human skin cancer [108]. Jervine was shown to block endogenous Shh signaling [103].

### 5.3. Hh Signaling Modulators

Recent reports suggest that vitamin D3, whose secretion can be facilitated by PTCH1, inhibits SMO signaling through direct binding to SMO [28]. Promising data indicate that the effect of tazarotene, a retinoid with retinoic acid receptor (RAR) beta/gamma specificity against BCC carcinogenesis is sustained after its withdrawal [110]. Curcumin, a spice for cooking, has also been reported to block Hh signaling-mediated carcinogenesis. Several natural products, including genistein, EGCG, and resveratrol have also been reported to affect Hh signaling in a mouse model of prostate cancer [111].

## 6. Hedgehog Signaling and Ovarian Cancer

Ovarian cancer is among the tumor entities where a tumorigenic activation of the Hh signaling pathway has been reported [68,112–115]. Aberrant activation of the Hh pathway is mediated through increased endogenous ligand-dependent expression of Hh or by ligand-independent mutations of PTCH, SMO and Su(Fu) in the pathway [116,117]. In a study by Liao *et al.*, overexpression of PTCH and Gli1 protein in ovarian cancers correlated with poor survival of the patients. The subcellular localization of the Hh signal protein may also be important. While Gli1 expression was mainly observed in the cytoplasm of ovarian epithelial tumors, a high level of Gli1 expression in invasive cancer samples was associated with scattered nuclear Gli1 immunoreactivity [71]. Furthermore, significantly elevated expression of Shh mRNA was observed in ovarian cancers compared to normal tissues and benign ovarian tumors, and was specific for particular histological types.

In addition, ectopic Gli1 overexpression in ovarian cancer cells increased cell proliferation, cell mobility, invasiveness and induced differentiation, identifying Gli1 expression as an independent prognostic marker [71]. Inhibition of the Hh pathway by treatment with 3-keto-*N*-(aminoethyl-aminocaproyl-dihydrocinnamoyl)-cyclopamine, a specific inhibitor of the Hh pathway, induced cancer cell apoptosis, suppressed cell growth, mobility and invasiveness, and also induced cancer cell dedifferentiation [71]. According to Chen *et al.*, the expression of Shh, Dhh, PTCH, SMO and Gli1 proteins was not observed in normal OSE, but was increased stepwise in benign, borderline and malignant neoplasms. In addition, immunoreactivity for Shh, Dhh, PTCH, SMO and Gli1 was strongly associated with cell proliferation assessed by Ki-67 [113]. Blocking the Hh signal using either cyclopamine or Gli1 siRNA resulted in remarkably decreased cell proliferation in ovarian carcinoma cells [113]. Treatment with cyclopamine induced not only G1 arrest but also apoptosis in ovarian carcinoma cells [113]. Among the Hh signal molecules, Dhh expression was correlated with poor prognosis of ovarian carcinoma patients [113]. As demonstrated by Ray *et al.*, the Hh pathway appears to be important in regulating growth of ovarian cancer spheroid-forming cells (SFCs) [112]. SFCs were treated with Hh agonists (Shh and Ihh) and the Hh inhibitor (cyclopamine) to detect changes in spheroid growth and survival. All four investigated ovarian cancer cell lines’ readily formed spheroids under non-adherent growth conditions, while the normal ovarian epithelial cell line failed to form SFCs. Moreover, compared to a control epithelial cell line, ovarian cancer cell lines demonstrated significant activation of the Hh pathway, determined by increased expression of intranuclear Gli1. Both Hh agonists showed significant increases in spheroid volume of at least 42-fold for Shh-treated cells, and 46-fold for Ihh-treated cells [112]. Regarding survival, SFCs were 30%–50% more resistant to cyclopamine than their corresponding monolayer cells [112]. Despite this resistance, inhibition of the Hh pathway with cyclopamine prevented further growth of SFCs. Taken together, the activation and inhibition of the Hh pathway showed significant association to enhanced growth and growth restriction, respectively [112].

Bhattacharya *et al.* investigated the effect of a specific Hh pathway blocker on clonal growth and proliferation of ovarian cancer cell, both *in vitro* and *in vivo*. Upregulation of the Hh pathway was observed in primary ovarian tumors and all of the human ovarian cancer cell lines tested. There, the authors demonstrated the Hh pathway to help maintaining the clonal growth of human ovarian carcinoma-derived cell lines. Moreover, the inhibition of Hh signaling by cyclopamine resulted in the inhibition of proliferation and clonal growth of all of the ovarian cancer cell lines *in vitro* and arrested the tumor growth *in vivo*. Furthermore, overexpression of PTCH1 inhibited the clonogenic capacity [68]. In a recent study, Yang *et al.* assessed Hh pathway activation in 34 ovarian epithelial tumor specimens by analyzing target gene expression by *in situ* hybridization, immunohistochemistry, and real-time PCR [114]. In contrast to previous reports, this group showed that the percentage of hedgehog signaling activation is low in ovarian cancer, suggesting that identification of tumors with activated hedgehog signaling activation will facilitate therapy with hedgehog signaling inhibitors. In addition, the authors found that even in the tumor with elevated expression of the hedgehog target genes Gli1 and PTCH1, expression of Shh is not correlated with Hh target gene expression, suggesting other mechanisms of hedgehog signaling activation in this particular cancer type. Moreover, the fact that hedgehog signaling activation was not associated with any particular subtypes of ovarian cancer implicates that the morphological classification of ovarian cancer may not reflect the molecular pathogenesis of this disease [114]. In line with this observation, Schmid *et al.* demonstrated that the expression of Hh pathway-related genes varied considerably among the investigated ovarian cancer tissue samples: more than half of the tumor samples showed Hh signaling or pathway activation, either by expression of transcription factors and Hh ligands, or by overexpression of Ihh/Shh and the PTCH1/PTCH2. In addition, gene expression heterogeneity between patient samples of the same histological type and grade was observed [115]. The conflicting results of the available studies might be due to the fact that different standards have been used to define Hh signaling activation in ovarian cancer, as already mentioned above, and that involvement of Hh signaling may occur only in specific tissues or cancer cell lines.

## 7. Targeting Hedgehog Pathway in Ovarian Cancer

In a recent study the potential role of Gli1 in resistance to anoikis—a cell death that occurs due to detachment of a cell from the extracellular matrix and hence a primary feature of a cell that undergoes metastasis—was demonstrated. Treatment of various ovarian cancer cells by different concentrations of diindolylmethane (DIM) reduced anoikis resistance in a concentration-dependent manner. Reduction in anoikis resistance is correlated with a decrease in the Gli1 expression. Shh treatment not only increased the expression of Gli1, but also blocked anoikis induced by DIM. To confirm the role of Gli1, cyclopamine was used, resulting in significantly reduced anoikis resistance in ovarian cancer cell lines associated with reduced expression of Gli1. Conversely, Shh treatment blocked cyclopamine-induced anoikis. Silencing Gli1 expression induced anoikis in the examined ovarian cancer cells. *In vivo* studies demonstrated that DIM- or cyclopamine-treated ovarian cancer cells under suspension culture conditions drastically lost their ability of tumor formation *in vivo* in mice [101]. McCann *et al.* investigated if inhibition of the Hh pathway could inhibit serous ovarian cancer growth. They used an *in vivo* preclinical model of serous ovarian cancer to characterize the antitumor activity of cyclopamine and IPI-926. Primary human serous ovarian tumor tissue was utilized to generate tumor xenografts in mice that were subsequently treated with cyclopamine or IPI-926 [107]. Both compounds showed significant antitumor activity as single agents [107]. When IPI-926 was used in combination with paclitaxel and carboplatin (T/C), no synergistic effect was observed. Maintaining treatment with IPI-926 after discontinuation of T/C continued to suppress tumor growth. Hh pathway activity was analyzed by RT-PCR to assess changes in Gli1 transcript levels. The results demonstrated that Gli1 expression in the stroma was specifically and significantly reduced within 24 hours of IPI-926 treatment, while expression of Gli1 in tumor cells was unaffected. However, prolonged (21 days) IPI-926 treatment led to significant decreases in both stromal and tumor Gli1 expression. These data suggest that prolonged inhibition of stromal Hh signaling could ultimately lead to decreased Hh signaling in tumor cells. The Gli1 mRNA expression data from the microdissected stroma of human serous ovarian cancer confirmed that the Hh pathway is active in human ovarian stroma, and demonstrated that elevated Gli1 mRNA levels are associated with shorter survival [107].

In a phase II, randomized, double-blind, placebo-controlled trial, vismodegib (GDC-0449) was tested to provide an estimate of efficacy in the setting of second or third complete remission in 104 ovarian cancer patients. Although numerical improvements were observed, a clinically meaningful improvement in progression-free survival for vismodegib *versus* placebo maintenance could not be demonstrated, and Hedgehog ligand-expression frequency was lower than expected [93]. One possible explanation for this result is that Hh ligand overexpression is associated with chemotherapy resistance that could prevent patients from achieving complete remission and, therefore, of being eligible for this study. Alternatively, it is also conceivable that Hedgehog ligand overexpression is actually associated with a lower probability of relapse, leading to a lower prevalence of Hh-positive ovarian cancer in this study population [93]. This report underlines the importance of an appropriate patient selection strategy for further studies.

## 8. Conclusions

The etiology of ovarian cancer is still among the poorest understood of common human malignancies. Even though 70%–80% of affected women achieve a complete clinical response to first line treatment, including surgery, the majority develops recurrent disease. The linkage of Hh signaling activation to various human cancers, including ovarian cancer, and the discovery of novel Hh signaling inhibitors provides promising options for developing novel targeted therapeutic strategies. Despite these advances, there are multiple goals to address for the future. The optimized use of Hh signaling antagonists accompanied by rational patient selection will increase the potential of Hh inhibitors as alternative molecularly targeted anticancer drugs. Well-designed clinical trials are necessary to evaluate the real potential of such pathway inhibitors. A rational patient selection will be based on tumor tissue-based expression or mutational testing with regard to the investigated Hh signaling inhibitor. Overexpression or pathway activation by gene mutations of members of the Hh signaling cascade might predict the efficacy of Hh signaling inhibitors and should therefore be tested and included as a translational research approach in each clinical trial. These steps could lead to the identification of predictive biomarkers to better identify patient subpopulations that will benefit from an Hh-targeted therapy. A better understanding of the underlying mechanisms of resistance is also urgently needed. Achieving these goals will be of paramount importance for designing targeted therapeutic strategies for ovarian cancer.

## Figures and Tables

**Table 1:** Overview about currently tested Hedgehog signaling inhibitors.

Inhibitor	*In vitro*/*in vivo* studies	References
Cyclopamine	*in vitro* and *in vivo* studies	[98–101]
KAAD cyclopamine	*in vitro* studies	[102]
Jervine	*in vitro* studies	[103]
Cyc T	*in vitro* and *in vivo* studies	[104]
Cur 61414	Phase I clinical trial	[88]
SANT 1,2,3,4	*in vitro* studies	[102]
Compound 5	*in vitro* studies	[105]
Compound Z	*in vitro* studies	[105]
GANT-58, -61	*in vitro* and *in vivo* studies	[106]
IPI 926	Phase I clinical trial and *in vivo* studies	[82,94,104,107]
GDC-0449 (Vismodegib)	Phase I and II clinical trial	[89–93]
BMS 833923 (XL139)	Phase I clinical trial	[95]
LDE 225	Phase I clinical trial	[96]
Vitamin D3	*in vitro* studies	[28]
Robotnikinin	*in vitro* studies	[108]
